# The Influence of Recent Climate Change on Tree Height Growth Differs with Species and Spatial Environment

**DOI:** 10.1371/journal.pone.0014691

**Published:** 2011-02-16

**Authors:** Yassine Messaoud, Han Y. H. Chen

**Affiliations:** Faculty of Natural Resources Management, Lakehead University, Thunder Bay, Canada; University College London, United Kingdom

## Abstract

Tree growth has been reported to increase in response to recent global climate change in controlled and semi-controlled experiments, but few studies have reported response of tree growth to increased temperature and atmospheric carbon dioxide (CO_2_) concentration in natural environments. This study addresses how recent global climate change has affected height growth of trembling aspen (*Populus tremuloides* Michx) and black spruce (*Picea mariana* Mill B.S.) in their natural environments. We sampled 145 stands dominated by aspen and 82 dominated by spruce over the entire range of their distributions in British Columbia, Canada. These stands were established naturally after fire between the 19^th^ and 20^th^ centuries. Height growth was quantified as total heights of sampled dominant and co-dominant trees at breast-height age of 50 years. We assessed the relationships between 50-year height growth and environmental factors at both spatial and temporal scales. We also tested whether the tree growth associated with global climate change differed with spatial environment (latitude, longitude and elevation). As expected, height growth of both species was positively related to temperature variables at the regional scale and with soil moisture and nutrient availability at the local scale. While height growth of trembling aspen was not significantly related to any of the temporal variables we examined, that of black spruce increased significantly with stand establishment date, the anomaly of the average maximum summer temperature between May-August, and atmospheric CO_2_ concentration, but not with the Palmer Drought Severity Index. Furthermore, the increase of spruce height growth associated with recent climate change was higher in the western than in eastern part of British Columbia. This study demonstrates that the response of height growth to recent climate change, i.e., increasing temperature and atmospheric CO_2_ concentration, did not only differ with tree species, but also their growing spatial environment.

## Introduction

Since the beginning of the Industrial Revolution in 1750, atmospheric carbon dioxide (CO_2_) concentration has increased steadily from 278 to 385 ppm. Global temperature has also increased by 0.6±0.2°C since the end of the Little Ice Age in 1880 [1], [2]. LaMarche *et al*. [3] were the first authors to report increase of tree growth in relation to atmospheric CO_2_ fertilization. Since then, various greenhouse and open top chamber experiments have shown higher growth of tree seedlings or short-lived trees with increased CO_2_ [4], [5]. These experiments have provided significant insights into the potential effects of global change, but the growing conditions and durations in controlled environments have led to concerns about their possible implications in the real world [6], [7]. In natural environments, tree growth has been shown to increase with recent atmospheric CO_2_ increase [8]–[10], while others found that increased tree growth is linked to global warming [11]–[13] or a combined effect of several factors such as atmospheric CO_2_ fertilization, global warming, and anthropogenic atmospheric nitrogen (N) deposition because of the inherent co-variation among these variables in natural environments [14].

One possible reason for these different findings may be the difference in tree growing environments, which has caused some tree species to become more sensitive to changes in their environment at their range limit [15], [16]. For example, several studies reported a greater response of tree growth related to climate change at the higher latitude or elevation compared with those located at their optimum range or at their lower latitude or elevation [17], [18]. To date, most studies on climate change effects on tree growth have taken place at a small spatial scale. Furthermore, potential different responses of individual species have largely been ignored in elevated CO_2_ research [19].

In North America, trembling aspen (*Populus tremuloides* Michx) and black spruce (*Picea mariana* Mill.) are the most widely distributed broadleaf and conifer species, respectively [20]. Both trembling aspen and black spruce can colonize burned areas immediately after wildfire. Trembling aspen reproduces through root suckering, whereas black spruce reproduces mostly from seeds after fire due to its semi-serotinous cores. In British Columbia, aspen is the most widely distributed deciduous species, whereas black spruce is generally seen in northern parts of the province [21]. Both species have wide ecological amplitudes, occurring with a range of soil moisture regimes from very dry to very moist and nutrient regimes from very poor to very rich.

The objective of this study was to examine the potential effects of recent global climate change and atmospheric CO_2_ on growth of trembling aspen and black spruce in British Columbia. While we expected height growth for both species is highly related to spatial environment over a large geographic area as reported in many other large-scale studies of tree height growth [14], [22]–[24], we hypothesized that (1) growth of these two species increases with increasing temperature and atmospheric CO_2_ concentration associated with recent climate change, and (2) growth response of the two species to temporal environment differs with spatial environment, more pronounced at more limiting temperature environments, e.g., high elevations or latitudes, because the effect of increased CO_2_ concentration on tree growth may be better facilitated by increased temperatures in these temperature limiting environments. To quantify global climate change effects on tree growth, previous studies have mostly used radial measurements [e.g.,[9],[25]]. In this study, we use tree height of dominant and co-dominant trees as a measure of tree growth. In comparison with radial growth, height of dominant and co-dominant trees is more strongly influenced by climate and site characteristics[26], [27]. Furthermore, height growth of dominant and co-dominant trees in a forest stand, e.g., site index (defined as the height of dominant and co-dominant trees at a reference age, usually 50 years at breast height), is a strong predictor for forest stand productivity and is less affected by stand density [23], [28], [29].

## Results

**Figure 1:**
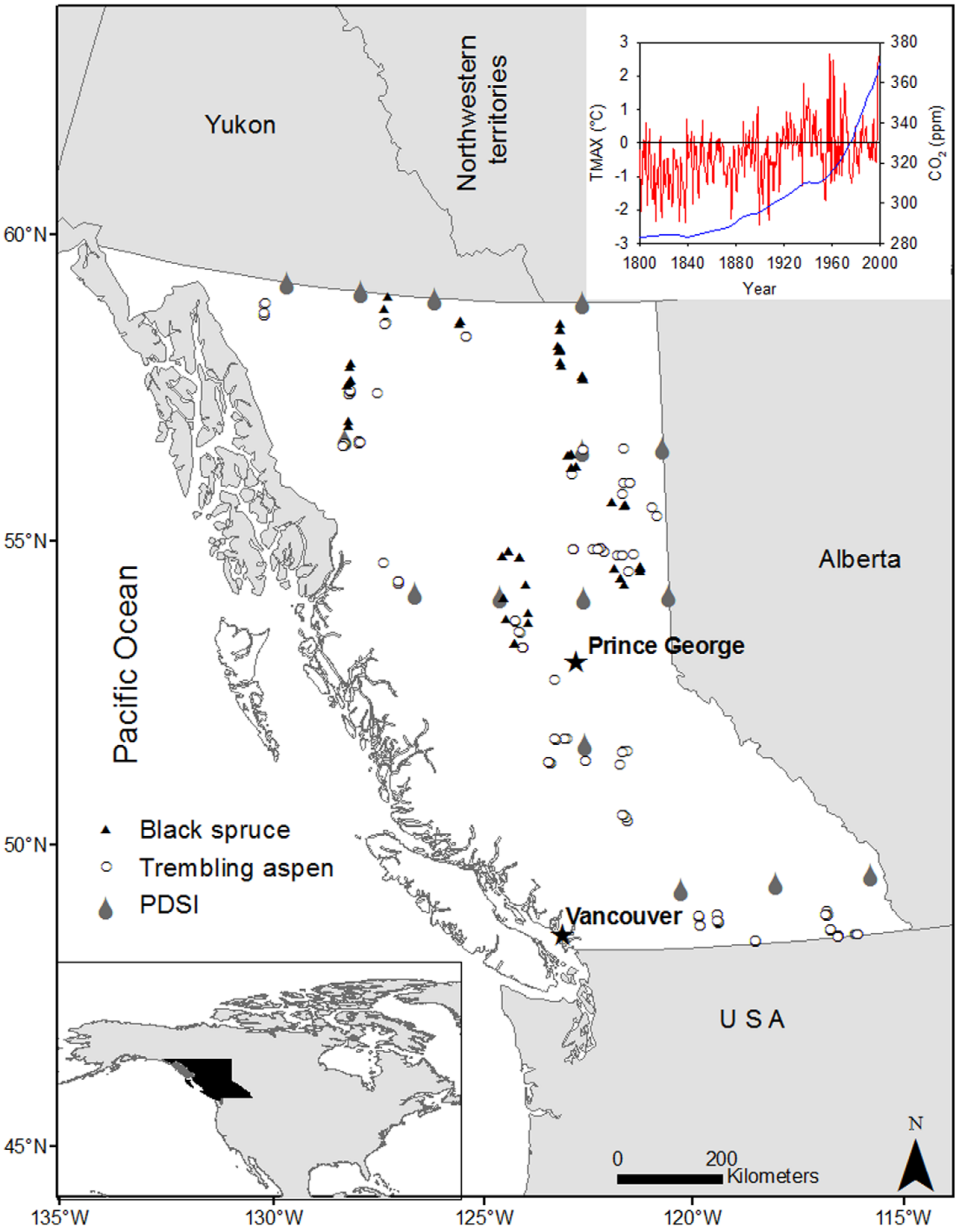
Locations of the sample plots for trembling aspen (white circle) and black spruce (black triangle) in British Columbia. Number of sample plots varies from one to twelve in each labeled location. Raindrops indicate the location of the PDSI. Insert shows evolution of temperature (anomaly of summer maximum May-August temperature, TMAX, °C) and atmospheric CO_2_ concentration (CO_2_, ppm) between year 1800 and 2000.

The sampled stands covered a wide range of climatic and site conditions in British Columbia (Table 1, Fig. 1). Site index varied between 6.0 to 35.1 m for trembling aspen and 8.3 to 25.2 m for black spruce, and stand ages ranged from 50 to 181 years for aspen and from 57 to 185 for black spruce, respectively.

**Table 1:** Characteristics (ranges) of study stands in British Columbia.

Species	Trembling aspen	Black spruce
No. of plots	145	82
Latitude (N)	49°35′–59°35′	54°12′–59°57′
Longitude (W)	115°30′–133°10′	120°47′–130°06′
Elevation (m)	380–1285	340–1170
Mean annual temperature (°C)^a^	−1.0–6.5	−2.5–3.1
Mean summer temperature (°C)	11.0–16.7	11.1–15.1
Mean summer precipitation (mm)	133–494	225–392
Mean annual precipitation (mm)^a^	295–1632	417–760
CO_2_ concentration (ppm)^b^	284.5–329.9	284.7–329.9
Stand age (years at breast height)	50–181	57–185
Height (m)	6.0–35.1	8.3–25.2
Site index (m)	5.52–30.70	4.69–14.04

a ClimateBC was used to derive spatial climatic attributes for the period of 1971–2000.

b http://cdiac.ornl.gov/ftp/trends/co2/maunaloa.co2; http://www.esrl.noaa.gov/gmd/ccgg/trends/ during the period between 1800–2008.

**Figure 2:**
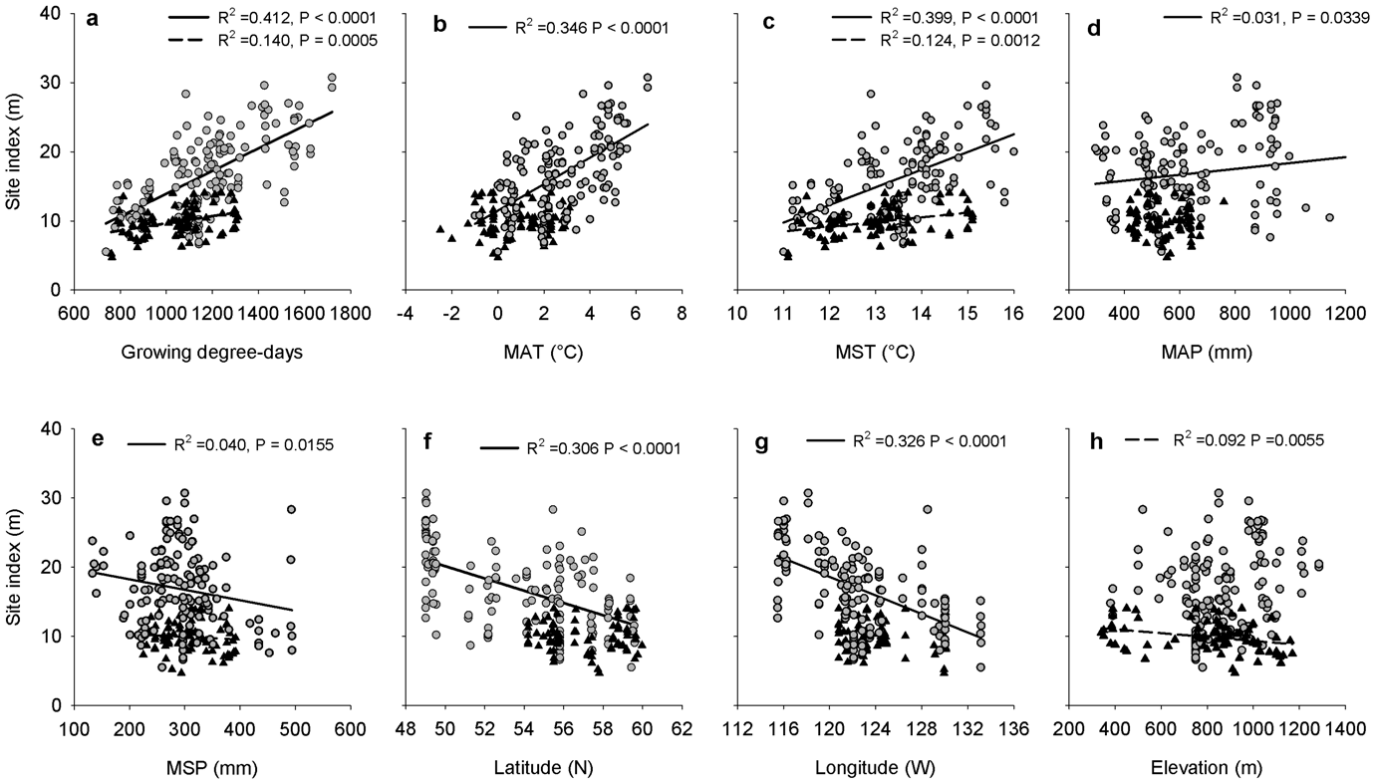
Site index (m) for the trembling aspen (grey circles and solid line for fitted regression when significant at a critical α = 0.05) and for black spruce (black triangle and dash line for fitted regression) in relation to spatial factors (*n* = 145 for aspen and 82 for spruce). (a) growing degree-days, (b) mean annual temperature (MAT, °C), (c) mean annual summer temperature (MST, °C), (d) mean annual precipitation (mm), (e) mean summer precipitation (mm), (f) latitude (°N), (g) longitude (°W), and (h) elevation (m).

**Figure 3:**
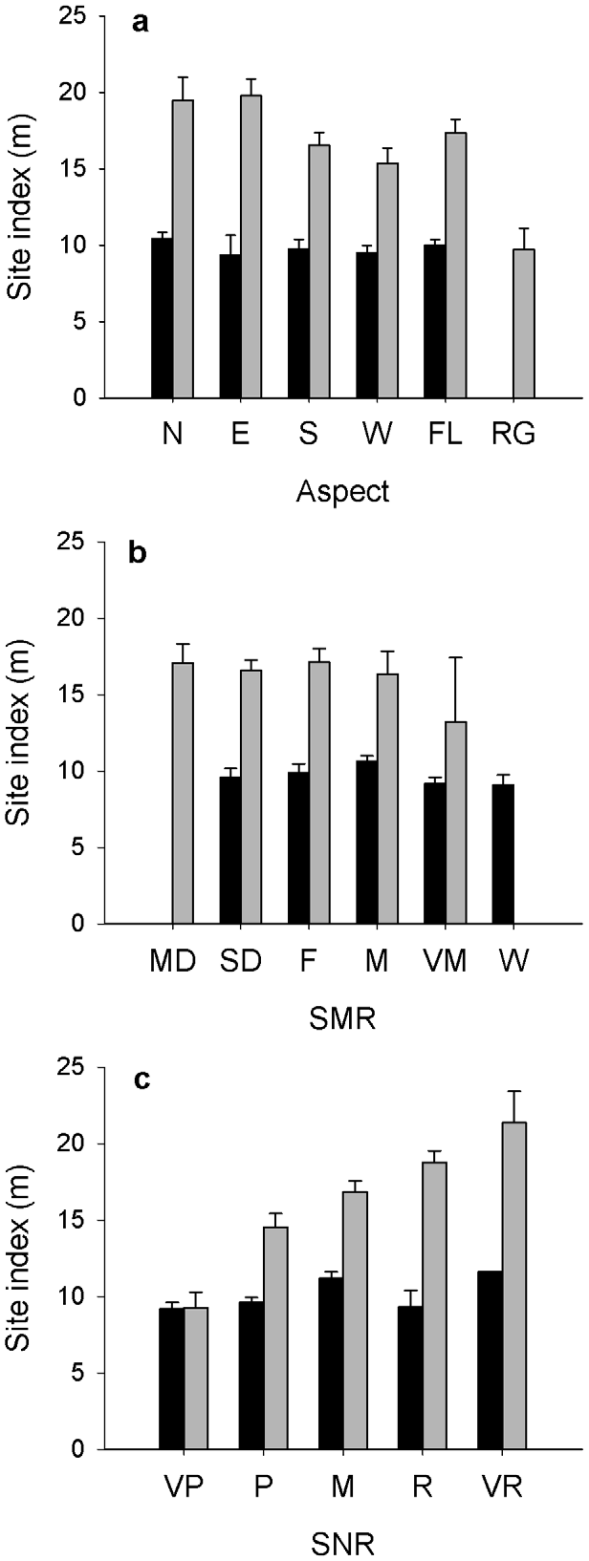
Site index (m) of trembling aspen (grey) and black spruce (black) in relation to local site condition. (a) aspect (RG, ridge; E, east; S, south aspect; W, west aspect; N, north aspect; and FL, flat), (b) soil moisture regime (SD, slightly dry; F, fresh; M, moist; VM, very moist; W, wet), and (c) soil nutrient regime (VP, very poor; P, poor; M, medium; R, rich; VR, very rich).

Aspen site index increased significantly with GDD, MAT, and MST (Figs. 2a–c), and marginally increased with MAP and marginally decreased with MSP (Figs. 2d and 2e). Similarly, spruce site index increased with GDD, MST (Figs. 2a and c), but it did not change with MAT, MAP or MSP (Figs. 2d and 2e). Aspen site index decreased with latitude and longitude, and did not change with elevation (Figs. 2f–h). By contrast, spruce site index decreased with elevation, and did not change with latitude or longitude (Figs. 2f–h). With changes in local site variables, the best site index of aspen was on the very rich, medium dry and fresh soil nutrient and moisture regimes respectively, on the north and east facing slope, while it was the lowest on the ridge slope position (Figs. 3a–c). The highest site index of spruce was on the north facing aspect, on the moist and medium soil moisture, and medium nutrient conditions (Figs. 3a–c).

General linear models showed that spatial variables collectively explained 51.7% of the variation of aspen site index, and 30.4% of the variation of spruce site index (Table 2). For aspen, the effect size by local site variables (SNR and aspect) was 0.171, and that by MST was 0.409. For spruce, the effect size by local site factor (SMR) was 0.128, and that by GDD was 0.176.

**Table 2:** Results of stepwise multiple regression model analyses between site index and climate and local site factors: growing-degree days, mean annual temperature (MAT, °C), mean summer temperature (MST, °C), mean annual precipitation (MAP, mm), and mean summer precipitation (MSP, mm), soil nutrient regime (SNR), aspect, and soil moisture regime (SMR).

Species	Source	df	*F*	*P*	Eta-squared
Trembling aspen	*Model* R^2^ = 0.517				
	MST	1	129.47	<0.0001	0.409
	SNR	4	8.00	<0.0001	0.101
	Aspect	6	3.70	0.0020	0.070
Black spruce	*Model* R^2^ = 0.304				
	GDD	1	19.28	<0.0001	0.176
	SMR	4	3.49	0.0113	0.128

**Figure 4:**
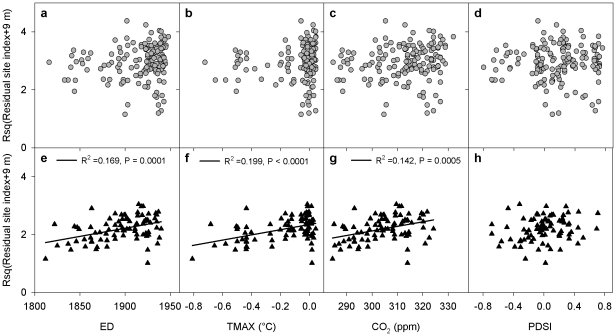
Residual site index (m) in relation to establishment date (ED, year), anomaly of summer maximum temperature (TMAX, °C), atmospheric CO_2_ concentration (CO_2_, ppm), and Palmer Drought Severity Index (PDSI). (a–d) for aspen (grey circle) and (e–h) for spruce (black triangle). Root squared transformation (Rsq) was applied to residual site index.

Simple regression analysis showed that residual site index resulting from removing the influence of spatial variables, was not significantly related to any temporal variables for aspen, whereas it significantly increased with ED, TMAX andCO_2_ concentration for spruce (Fig. 4). Among individual temporal variables, ED, TMAX, and CO_2_ explained 16.9, 19.9, and 14.2% of the variation of spruce residual site index, respectively (Fig. 4).

**Figure 5:**
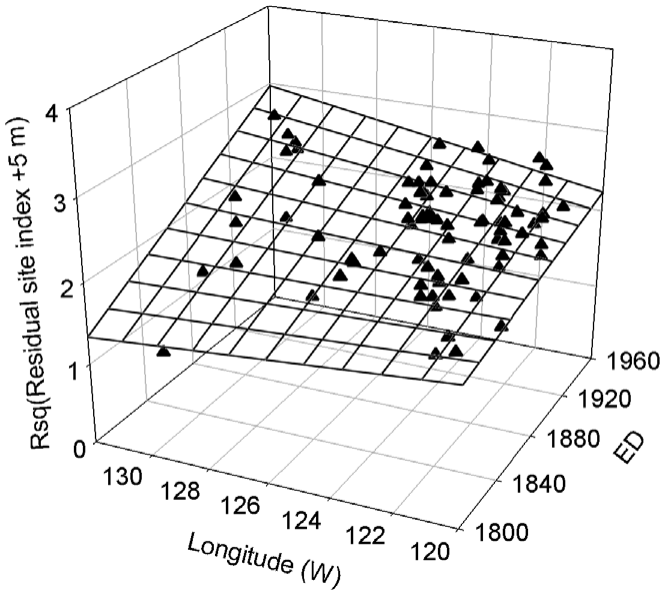
The effect of establishment date (ED, year) and geographic locations on residual site index for spruce (*n* = 82).

Multiple regression analysis showed that, for spruce, the residual site index increased with ED and decreased eastward, but the increase associated with ED was faster in western than eastern part of the province (Table 3, Fig. 5). For aspen, the residual site index was not significantly related to ED, latitude, longitude, elevation, or their interactions in the full model, and various combinations of manual variable selection did not yield any models better than the null model (intercept only) based on AIC.

**Table 3:** Results of stepwise multiple regression analyses between residual site index, establishment date (ED), geographic location (latitude, longitude and elevation), and their interactions.

Species	Source	*Coefficient*	*F*	*P*
Black spruce	*Model* R^2^ = 0.184			
	Longitude	0.066532	7.20	0.0089
	Longitude × ED	−0.000048	18.13	<0.0001

Only significant variables (α<0.05) are retained using backward stepwise procedure.

## Discussion

### Spatial environment

At the spatial scale, height growth of both species responded positively to temperature related variables. This finding was consistent with height growth studies located in middle to high latitudes in North America [30], [31] or in Europe [32], [33]. Warmer temperature and longer growing season, associated with lower latitudes and elevations, promote photosynthesis and carbohydrate allocation to the stem [34]. The slower height growth of aspen in the northwestern part of the province, i.e., higher longitudes, may be a result of shorter growing season because of the frequent period of cloudiness in the western part of the province [35]. As expected, our analysis also showed that strong influences of local site condition, measured by slope position, soil moisture and nutrient regimes, is a strong predictor for tree height growth as found in other site quality studies [22], [36].

Both species responded weakly to precipitation. Aspen height growth had a marginally positive and negative relationship with mean annual and summer precipitation, respectively, whereas spruce height growth was not significantly related to either precipitation variable. The weak or absence of a relationship between height growth and precipitation appears to support previous findings that tree growth in northern climates is driven by temperature than water availability [37]. Since spruce is located in the northern part of the province, where water evaporation is low [21], and the species occurs mostly on sites with no or minimum soil moisture limitations in western North America [38], [39], additional water input may reduce growth. This is because in colder environments with low soil evaporation, water surplus increases the frequency of flooding, which reduces the rate of photosynthesis due to the stomata closure and reduction of root activity [40]. Multiple regression analysis further confirmed that precipitation has no effect on height growth of either species, suggesting that precipitation is not a limiting factor or any potential influences of precipitation on height growth is coupled with temperature related variables, i.e., MST for aspen and GDD for spruce (Table 2).

Consistent with the common thought [20], [41], aspen, a fast growing species, appeared to be more sensitive to the spatial environmental change (climate and local site condition) than spruce, in terms of the magnitude of their responses to the temperature related variables. Alternatively, their responses reflect the geographic range associated climatic variations in the studied province, i.e., a bigger range sampled in independent variables increase the statistical strength of their association with the dependent variable.

### Temporal environment

Atmospheric CO_2_ concentration is often mentioned as being responsible for recent increase in tree growth [8], [9], [42], whereas others attribute tree growth increase to the effect of increased temperature during the growing season [12], [13]. Our study indicated that the recent global warming coupled with increased atmospheric CO_2_ concentration, and to a lesser extent of nitrogen deposition as it is low compared with other regions [43], is accountable for increased height growth during the period of 160 years for spruce. However, neither recent climate change variables (TMAX, PDSI) nor atmospheric CO_2_ had affected aspen height growth at the scale of the entire province. Can the different responses of the two species be a result of their genetic differences or different strategies to adapt or acclimate to the recent increase in temperature and CO_2_? Potential mechanisms associated with the different responses among species have not been well understood since few studies have compared the responses of different plant species to climate warming and elevated CO_2_ [19]. Alternative to the fact that their genetic differences have dictated their difference responses to climate change and elevated CO_2_, the different responses of the two species could be a result of the different geographic ranges studied. In British Columbia, spruce is a northern species, well adapted to cold climates, while aspen is one of the widest distributed species [21]. The climate change coupled with increased CO_2_ may have a positive effect on tree growth in northern rather than southern environments [12], [13], [16].

The height growth response to climate change differed with geographic locations for spruce with a greater height growth response at the western than at the eastern parts of the province. In the western part, spruce is located in higher elevations with cooler summer temperatures and higher water availability compared with the western part [44]. The better growth response of spruce in the western part of our sample area appears to be a result of the interplay of increased temperature and CO_2_ in the environment where growing season temperature is more limited than water availability.

### Conclusions

This study attempted to relate tree height growth of trembling aspen and black spruce to the recent global climate change in their natural environments in entire British Columbia. As expected, height growth of both species was positively related to temperature variables at the regional scale and with soil moisture and nutrient availability at the local scale. While height growth of trembling aspen was not significantly related to any of the temporal variables we examined, that of black spruce increased significantly with temporal variables including stand establishment date, the anomaly of the average maximum summer temperature between May-August, and atmospheric CO_2_ concentration, but not with the Palmer Drought Severity Index. As hypothesized, the increase in spruce height growth associated with recent climate change differed with its geographic location, i.e., a higher increase in the western than in eastern part of the study area. However, the higher increase in the western part was apparently a result of the interplay of increased temperature and CO_2_ in the environment where growing season temperature is more limited than water availability. The mechanisms for the different responses to climate change of the two species are, however, not well understood.

## Materials and Methods

### Study area

This study was designed to capture the widest range of climate, soil moisture, and soil nutrient regimes in which the study species can naturally establish in the province (Table 1 and Fig. 1) [44]. The study area is located between 49°N and 60°N and 115°30′W and 134°W with elevation ranging from 340 to 1285 m above sea level in British Columbia, Canada. Wildfire is a common stand-replacing disturbance in the study area. Both species studied grow on a variety of soils, ranging from shallow and rocky to deep loamy sands and heavy clays; black spruce can also grow on organic soils. Depending on regional climate, aspen occurs in co-dominance with black spruce, balsam poplar (*Populus balsamifera* L.) white spruce (*Picea glauca* (Moench) Voss), hybrid spruce (*Picea engelmannii* Parry *ex* Engelm. x *Picea glauca* (Moench) Voss), subalpine fir (*Abies lasiocarpa* (hook.) nutt.), lodgepole pine (*Pinus contorta* Dougl. ex. Loud.), white birch (*Betula papyrifera* Marsh.), and Douglas-fir (*Pseudotsuga menziesii* var. *glauca*). Black spruce is commonly associated with white spruce, lodgepole pine and trembling aspen.

### Sampling design

We sampled 145 and 82 stands for aspen and spruce, respectively. All sampled stands were naturally established after wildfire, unmanaged, and without a history of suppression or damage. These stands are of variable ages (Table 1), and as such, for a given period of growth, e.g., ages between 0 to 50 years, stands had experienced a wide range of historical growing conditions of atmospheric CO_2_ and temperature associated with global climate change.

Within each sampled stand, a plot of area 0.04 ha was established. Soil and vegetation were described according to Luttmerding *et al*. [45]. Topographic maps were used to determine the latitude and longitude of each plot, elevation was measured with a Thommen pocket altimeter, and aspect was measured with a Suunto pocket compass. Soil moisture (SMR) and nutrient regimes (SNR) were estimated using regional ecosystem field guides [46]. Within a plot, three or four trees of largest diameters with no visible growth abnormalities were sampled. For each tree selected, stem discs were cut at 30 cm above the root collar, and at 1-m intervals to the top of the tree. Stunted trees had stem discs cut at 30 cm above the root collar and at 50-cm intervals to the top of the tree [22]. In the laboratory, each disc was transversely cut with a sharp knife and zinc oxide powder was added to make the rings clearly visible. With a microscope, rings were counted in two directions until the same count was obtained. Particular attention was paid to abrupt changes in radial increment indicating possible suppression or damages. Raw stem analysis data were adjusted using Carmean's algorithm to calculate tree height corresponding to the age at each sectioned disc [47]. An average height growth curve was then computed from three sampled trees for each study plot using Richards' three-parameters equation [29], [48]. For each plot, site index at the reference age of 50 years at breast height was then calculated from the fitted equation.

### Spatial environment

For each sample plot, spatial climate attributes were derived from its spatial coordinates, latitude, longitude, and elevation by using the ClimateBC [49], [50]. These data were estimated by extrapolation of climate data from the meteorological stations located in the region for the period 1971–2000. The climate attributes included sum of growing degree-days >5°C (GDD), mean annual temperature (MAT, °C), mean summer temperature (June-August, MST, °C), mean annual precipitation (MAP, mm), and mean summer precipitation (MSP, mm, i.e., total precipitation May to September inclusive). To examine the spatial climate influence on site index, the averages of the above climate attributes for the period of 1971–2000 were used.

Local site conditions including aspect, SMR, and SNR have a profound influence on tree height growth [22]. For each sampled plot, aspect was classified as ridge, east (azimuth 45–135°), south (azimuth 135–225°), west (azimuth 229–315°), north (azimuth 315-45°), or flat; SMR as very dry, moderately dry, slightly dry, fresh, moist, or very moist, and SNR as very poor, poor, medium, rich, or very rich.

### Climate change and atmospheric CO_2_ concentration data

To assess the effects of climate change and CO_2_ on site index, we used reconstructions of the anomaly of the average maximum summer temperature between May-August (TMAX) and the Palmer Drought Severity Index (PDSI) for western Canada, provided by the National Oceanic and Atmospheric Administration (NOAA; http://www.ncdc.noaa.gov/paleo/recons.html) as predictors. The TMAX records were derived from climate reconstruction in the Canadian Rockies (Alberta, 52° 15′N and 117° 15W) for the period of 950 AD-1994 [51] to represent the temperature condition for the whole study area. The reference period for the anomaly values in the climate reconstruction is 1900–1980. The PDSI value is based on the soil water budget model and indicates cumulative long-term dry (negative) and wet (positive) weather conditions for a period spanning from 750 to 1990 (http://www.ncdc.noaa.gov/paleo/pdsidata.html) [52], [53]. Since historical PDSI data were available in 2.5×2.5° latitude and longitude grids (Fig. 1), we chose the nearest PDSI for each sampled plot. Historical global atmospheric CO_2_ concentration data were obtained from Antarctic ice cores and from Mauna Loa observatory (Hawaii, USA) covering 1006 AD-1978 AD (http://cdiac.esd.ornl.gov/trends/co2/lawdome.html) and 1958–2008 (http://www.esrl.noaa.gov/gmd/ccgg/trends), respectively.

Since site index refers to the cumulative height growth above breast-height (1.3 m) for a period of 50 years, we used the average of temporal climate conditions and CO_2_ concentration of the corresponding period of tree growth to examine temporal influence on site index.

### Statistical analyses

We first tested the effect of the spatial variables on the site index; we then examined the effect of the temporal variables on residual site index, allowing for the control of the influence of spatial variables before including the temporal variables [54]. For each plot, spatial environment was represented by climate variables, i.e., GDD, MAT, MST, MAP, and MSP, and site variables, i.e., aspect, SMR, and SNR. General linear model was used to evaluate the effects of spatial variables on site index. Both complete and various manually selected stepwise models were evaluated to derive the final spatial models, in which all selected independent variables were significant at *α* = 0.05. Overall model significance and goodness-of-fit was judged using the Likelihood Ratio Statistic and assessing change in Akaike's Information Criterion (AIC) scores. A change in AIC of >2 is considered a substantial change in the descriptive ability between models [55]. Model goodness-of-fit was interpreted from *R* ^2^ and by evaluating the effect-size measures of the predictor variables retained in the final models. The effect size of the predictor variables was calculated using Eta-squared, which represents the proportion of total sum of squares explained by each predictor variable in the model [56].

The effect of individual temporal variables (establishment date, TMAX, CO_2_, and PDSI) on residual site index was first examined by simple regression analysis. To achieve normality and homogeneity of variance, root square transformation of residual site index was applied by adding a positive integer, i.e., 9 for aspen and 5 for spruce as the smallest value of residual site index was −8.5 and −4.1 for aspen and spruce, respectively.

To test the second hypothesis, we used multiple regressions to relate the transformed residual site index to establishment date (ED), which correlates strongly with other temporal variables (Table S1), and geographic locations (latitude, longitude and elevation), which correlate strongly with spatial climate variables (Table S2), by including interaction terms of ED × latitude, ED × longitude, and ED × elevation. ED was calculated as the mean of the establishment dates of the three sampled trees. The response of the residual site index to other temporal variables and their interactions with geographic locations were also examined, but resulted in much weaker relationships. Similar to the analysis for spatial environment, we evaluated both complete and stepwise models, and the final models were selected based on AIC and a significance level of *α* = 0.05 for the selected independent variables. All analyses were performed with SYSTAT 12 [57].

## Supporting Information

Table S1 Pearson's correlation between establishment date (ED, year), the anomaly of the average maximum summer temperature between May-August (TMAX), atmospheric CO_2_ concentration (ppm), and the Palmer Drought Severity Index (PDSI).
(0.03 MB DOC)

Table S2 Pearson's correlation between geographic locations (latitude, longitude and elevation) and spatial climate variables, sum of growing degree-days >5°C (GDD), mean annual temperature (MAT, °C), mean summer temperature (June-August, MST, °C), mean annual precipitation (MAP, mm), and mean summer precipitation (MSP, mm, i.e., total precipitation May to September inclusive).
(0.03 MB DOC)

## References

[1] Fantin N, Morin H (2002). Comparative juvenile growth of two successive generations of black spruce from seeds after the boreal forest fire in Quebec.. Canadian Journal of Forest Research.

[2] Boisvenue C, Running SW (2006). Impacts of climate change on natural forest productivity - evidence since the middle of the 20th century.. Global Change Biology.

[3] Lamarche VC, Graybill DA, Fritts HC, Rose MR (1984). Increasing atmospheric carbon-dioxide - Tree-ring evidence for growth enhancement in natural vegetation.. Science.

[4] Asshoff R, Zotz G, Korner C (2006). Growth and phenology of mature temperate forest trees in elevated CO_2_.. Global Change Biology.

[5] Moore DJP, Aref S, Ho RM, Pippen JS, Hamilton JG (2006). Annual basal area increment and growth duration of *Pinus taeda* in response to eight years of free-air carbon dioxide enrichment.. Global Change Biology.

[6] Gifford RM (2004). The CO_2_ fertilising effect - does it occur in the real world? The International Free Air CO_2_ Enrichment (FACE) Workshop: Short- and long-term effects of elevated atmospheric CO_2_ on managed ecosystems, Ascona, Switzerland, March 2004.. New Phytologist.

[7] Saxe H, Ellsworth DS, Heath J (1998). Tree and forest functioning in an enriched CO_2_ atmosphere.. New Phytologist.

[8] Soulé PT, Knapp PA (2006). Radial growth rate increases in naturally occurring ponderosa pine trees: a late-20th century CO_2_ fertilization effect?. New Phytologist.

[9] Wang GG, Chhin S, Bauerle WL (2006). Effect of natural atmospheric CO_2_ fertilization suggested by open-grown white spruce in a dry environment.. Global Change Biology.

[10] Koutavas A (2008). Late 20th century growth acceleration in Greek firs (*Abies cephalonica*) from Cephalonia Island, Greece: A CO_2_ fertilization effect?. Dendrochronologia.

[11] Penuelas J, Hunt JM, Ogaya R, Jump AS (2008). Twentieth century changes of tree-ring delta C-13 at the southern range-edge of *Fagus sylvatica*: increasing water-use efficiency does not avoid the growth decline induced by warming at low altitudes.. Global Change Biology.

[12] Graumlich LJ (1991). Sub-alpine tree growth, climate, and increasing CO_2_ - An assessment of recent growth trends.. Ecology.

[13] Wilmking M, Juday GP, Barber VA, Zald HSJ (2004). Recent climate warming forces contrasting growth responses of white spruce at treeline in Alaska through temperature thresholds.. Global Change Biology.

[14] Pinto PE, Gegout JC, Herve JC, Dhote JF (2008). Respective importance of ecological conditions and stand composition on *Abies alba* Mill. dominant height growth.. Forest Ecology and Management.

[15] Loehle C (2000). Forest ecotone response to climate change: sensitivity to temperature response functional forms.. Canadian Journal of Forest Research.

[16] Reich PB, Oleksyn J (2008). Climate warming will reduce growth and survival of Scots pine except in the far north.. Ecology Letters.

[17] Srur AM, Villalba R, Villagra PE, Hertel D (2008). Influences of climatic and CO2 concentration changes on radial growth of *Nothofagus pumilio* in Patagonia.. Revista Chilena de Historia Natural.

[18] Kellomäki S, Kolstrom M (1994). The Influence of Climate-Change on the Productivity of Scots Pine, Norway Spruce, Pendula Birch and Pubescent Birch in Southern and Northern Finland.. Forest Ecology and Management.

[19] Bradley KL, Pregitzer KS (2007). Ecosystem assembly and terrestrial carbon balance under elevated CO2.. Trends in Ecology & Evolution.

[20] Burns, RM, Honkala, BH (1990). Silvics of North America..

[21] Klinka K, Worrall J, Skoda L, Varga P, Krajina VJ (2000). The distribution and synopsis of ecological and silvical characteristics of tree species of British Columbia's forests..

[22] Chen HYH, Krestov PV, Klinka K (2002). Trembling aspen site index in relation to environmental measures of site quality at two spatial scales.. Canadian Journal of Forest Research.

[23] Chen HYH, Klinka K, Kabzems RD (1998). Site index, site quality, and foliar nutrients of trembling aspen: relationships and predictions.. Canadian Journal of Forest Research.

[24] Klinka K, Chen HYH (2003). Potential productivity of three interior subalpine forest tree species in British Columbia.. Forest Ecology and Management.

[25] Lapointe-Garant MP, Huang JG, Gea-Izquierdo G, Raulier F, Bernier P (2010). Use of tree rings to study the effect of climate change on trembling aspen in Quebec.. Global Change Biology.

[26] Klinka K, Wang Q, Carter RE, Chen HYH (1996). Height growth-elevation relationships in subalpine forests of interior British Columbia.. Forestry Chronicle.

[27] Hamel B, Belanger N, Pare D (2004). Productivity of black spruce and Jack pine stands in Quebec as related to climate, site biological features and soil properties.. Forest Ecology and Management.

[28] Monserud RA (1984). Height growth and site index curves for Inland Douglas-fir based on stem analysis data and forest habitat type.. Forest Science.

[29] Chen HYH, Klinka K, Kabzems RD (1998). Height growth and site index models for trembling aspen (*Populus tremuloides*) in northern British Columbia.. Forest Ecology and Management.

[30] Nigh GD (2006). Impact of climate, moisture regime, and nutrient regime on the productivity of Douglas-Fir in coastal British Columbia, Canada.. Climatic Change.

[31] Nigh GD, Ying CC, Qian H (2004). Climate and productivity of major conifer species in the interior of British Columbia, Canada.. Forest Science.

[32] Seynave I, Gegout JC, Herve JC, Dhote JF (2008). Is the spatial distribution of European beech (*Fagus sylvatica* L.) limited by its potential height growth?. Journal of Biogeography.

[33] Fries A, Ruotsalainen S, Lindgren D (1998). Effects of temperature on the site productivity of Pinus sylvestris and lodgepole pine in Finland and Sweden.. Scandinavian Journal of Forest Research.

[34] Miyamoto Y, Griesbauer HP, Green DS (2010). Growth responses of three coexisting conifer species to climate across wide geographic and climate ranges in Yukon and British Columbia.. Forest Ecology and Management.

[35] Dai A, Trenberth KE, Karl TR (1999). Effects of clouds, soil moisture, precipitation, and water vapor on diurnal temperature range.. Journal of Climate.

[36] Pinno BD, Pare D, Guindon L, Belanger N (2009). Predicting productivity of trembling aspen in the Boreal Shield ecozone of Quebec using different sources of soil and site information.. Forest Ecology and Management.

[37] Bloom AJ, Chapin FS, Mooney HA (1985). Resource limitation in plants - An economic analogy.. Annual Review of Ecology and Systematics.

[38] Wirth C, Lichstein JW, Dushoff J, Chen A, Chapin FS (2008). White spruce meets black spruce: Dispersal, postfire establishment, and growth in a warming climate.. Ecological Monographs.

[39] Viereck LA, Little EL (2007). Alaska trees and shrubs..

[40] Kozlowski TT, Pallardy SG (1997). Physiology of woody plants..

[41] Leonelli G, Denneler B, Bergeron Y (2008). Climate sensitivity of trembling aspen radial growth along a productivity gradient in northeastern British Columbia, Canada.. Canadian Journal of Forest Research.

[42] Voelker SL, Muzika RM, Guyette RP, Stambaugh MC (2006). Historical CO_2_ growth enhancement declines with age in *Quercus* and *Pinus*.. Ecological Monographs.

[43] Holland EA, Dentener FJ, Braswell BH, Sulzman JM (1999). Contemporary and pre-industrial global reactive nitrogen budgets.. Biogeochemistry.

[44] Meidinger DV, Pojar J (1991). Ecosystems of British Columbia..

[45] Luttmerding H, Demarshi DA, Lea EC, Meidinger D (1990). Describing ecosystems in the field. 2nd edition..

[46] Green RN, Klinka K (1994). A field guide for site identification and interpretation for the Vancouver Forest Region..

[47] Dyer ME, Bailey RL (1987). A test of 6 methods for estimating true heights from stem analysis data.. Forest Science.

[48] Nigh GD, Krestov PV, Klinka K (2002). Height growth of black spruce in British Columbia.. Forestry Chronicle.

[49] Hamann A, Wang TL (2005). Models of climatic normals for genecology and climate change studies in British Columbia.. Agricultural and Forest Meteorology.

[50] Wang T, Hamann A, Spittlehouse DL, Aitken SN (2006). Development of scale-free climate data for western Canada for use in resource management.. International Journal of Climatology.

[51] Luckman BH, Wilson RJS (2005). Summer temperatures in the Canadian Rockies during the last millennium: a revised record.. Climate Dynamics.

[52] Piovesan G, Biondi F, Di Filippo A, Alessandrini A, Maugeri M (2008). Drought-driven growth reduction in old beech (*Fagus sylvatica* L.) forests of the central Apennines, Italy.. Global Change Biology.

[53] Cook ER, Woodhouse CA, Eakin CM, Meko DM, Stahle DW (2004). Long-term aridity changes in the western United States.. Science.

[54] Graham MH (2003). Confronting multicollinearity in ecological multiple regression.. Ecology.

[55] Chatterjee S, Hadi A (2006). Regression analysis by example..

[56] Howell CD (2007). Statistical methods for psychology..

[57] SYSTAT (2007). Systat 12 for Windows..

