# Supplementary material for: The Influence of Recent Climate Change on Tree Height Growth Differs with Species and Spatial Environment
Source: PLoS One. 2011 Feb 16;6(2):e14691. doi: 10.1371/journal.pone.0014691 (PMC3040169; doi:10.1371/journal.pone.0014691)
Supplement: Table S1 — Pearson's correlation between establishment date (ED, year), the anomaly of the average maximum summer temperature between May-August (TMAX), atmospheric CO2 concentration (ppm), and the Palmer Drought Severity Index (PDSI). (0.03 MB DOC) [file pone.0014691.s001.doc]

**Table S1.** Pearson's correlation between establishment date (ED, year), the anomaly of the average maximum summer temperature between May-August (TMAX), atmospheric CO_2_ concentration (ppm), and the Palmer Drought Severity Index (PDSI). Correlation is at *P* < 0.001 (***).

| Species | Variable | ED | TMAX | CO_2_ |
| --- | --- | --- | --- | --- |
| Trembling aspen | ED |  |  |  |
|  | TMAX | 0.947*** |  |  |
|  | CO_2_ | 0.976*** | 0.868*** |  |
|  | PDSI | 0.805*** | 0.685*** | 0.846*** |
| Black spruce | ED |  |  |  |
|  | TMAX | 0.968*** |  |  |
|  | CO_2_ | 0.984*** | 0.925*** |  |
|  | PDSI | 0.635*** | 0.577*** | 0.692*** |
