# Supplementary material for: The Influence of Recent Climate Change on Tree Height Growth Differs with Species and Spatial Environment
Source: PLoS One. 2011 Feb 16;6(2):e14691. doi: 10.1371/journal.pone.0014691 (PMC3040169; doi:10.1371/journal.pone.0014691)
Supplement: Table S2 — Pearson's correlation between geographic locations (latitude, longitude and elevation) and spatial climate variables, sum of growing degree-days >5°C (GDD), mean annual temperature (MAT, °C), mean summer temperature (June-August, MST, °C), mean annual precipitation (MAP, mm), and mean summer precipitation (MSP, mm, i.e., total precipitation May to September inclusive). (0.03 MB DOC) [file pone.0014691.s002.doc]

| Species | Variable | GDD | MAT | MST | MAP | MSP |
| --- | --- | --- | --- | --- | --- | --- |
| Trembling aspen | Latitude | -0.783*** | -0.922*** | -0.754*** | -0.068ns | 0.512*** |
|  | Longitude | 0.883*** | 0.770*** | 0.888*** | 0.055ns | -0.323** |
|  | Elevation | 0.144ns | 0.337** | 0.124ns | -0.102ns | -0.411*** |
| Black spruce | Latitude | 0.140ns | -0.96*** | 0.278ns | -0.759*** | -0.066ns |
|  | Longitude | 0.610*** | 0.460*** | 0.541*** | 0.281ns | 0.684*** |
|  | Elevation | -0.728*** | 0.452*** | -0.795*** | 0.655*** | 0.338ns |

Correlation is at *P* < 0.001 (***), < 0.01 (**), < 0.05 (*), or not significant (ns).
